# SEVA-Cpf1, a CRISPR-Cas12a vector for genome editing in cyanobacteria

**DOI:** 10.1186/s12934-022-01830-4

**Published:** 2022-05-28

**Authors:** Sara Baldanta, Govinda Guevara, Juana María Navarro-Llorens

**Affiliations:** Department of Biochemistry and Molecular Biology, Universidad Complutense de Madrid, 28040 Madrid, Spain

**Keywords:** SEVA vectors, Cyanobacteria, Genetic tools, CRISPR-Cpf1

## Abstract

**Background:**

Cyanobacteria are photosynthetic autotrophs that have tremendous potential for fundamental research and industrial applications due to their high metabolic plasticity and ability to grow using CO_2_ and sunlight. CRISPR technology using Cas9 and Cpf1 has been applied to different cyanobacteria for genome manipulations and metabolic engineering. Despite significant advances with genome editing in several cyanobacteria strains, the lack of proper genetic toolboxes is still a limiting factor compared to other model laboratory species. Among the limitations, it is essential to have versatile plasmids that could ease the benchwork when using CRISPR technology.

**Results:**

In the present study, several CRISPR-Cpf1 vectors were developed for genetic manipulations in cyanobacteria using SEVA plasmids. SEVA collection is based on modular vectors that enable the exchangeability of diverse elements (e.g. origins of replication and antibiotic selection markers) and the combination with many cargo sequences for varied end-applications. Firstly, using SEVA vectors containing the broad host range RSF1010 origin we demonstrated that these vectors are replicative not only in model cyanobacteria but also in a new cyanobacterium specie, *Chroococcidiopsis* sp., which is different from those previously published. Then, we constructed SEVA vectors by harbouring CRISPR elements and showed that they can be easily assimilated not only by conjugation, but also by natural transformation. Finally, we used our SEVA-Cpf1 tools to delete the *nblA* gene in *Synechocystis* sp. PCC 6803, demonstrating that our plasmids can be applied for CRISPR-based genome editing technology.

**Conclusions:**

The results of this study provide new CRISPR-based vectors based on the SEVA (Standard European Vector Architecture) collection that can improve editing processes using the Cpf1 nuclease in cyanobacteria.

**Supplementary Information:**

The online version contains supplementary material available at 10.1186/s12934-022-01830-4.

## Background

Nowadays, prokaryotes are broadly used in the production of valuable compounds for industrial and pharmacological purposes [1]. Cyanobacteria are attractive microorganisms as photoautotrophic microbial chassis because of their capability to grow in the presence of CO_2_ and sunlight. Therefore, not only does cyanobacterial metabolism not require expensive feedstock but it also reduces greenhouse emissions and decreases dependence on petroleum-based products [2]. Additionally, cyanobacteria are also an attractive model organism for physiological and ecological research, for instance studies into their metabolic responses to different abiotic stresses or their photosynthetic apparatus [3].

Commonly used cyanobacteria such as *Synechococcus elongatus* UTEX 2973, *Synechococcus* sp. PCC 7002, *Synechococcus elongatus* PCC 7942 and *Synechocystis* sp. PCC 6803 (hereafter, *Synechococcus* 2973, *Synechococcus* 7002, *Synechococcus* 7942 and *Synechocystis* 6803) have been engineered for industrial applications. Recently, newly discovered strains with faster growth e.g. *Synechococcus* PCC 11,801 and PCC 11,901 (hereafter, *Synechococcus* 11,801 and *Synechococcus* 11,901) are also becoming popular for biotechnological purposes [4, 5]. To date, cyanobacteria have been exploited to convert CO_2_ into a wide range of valuable products, for instance: biofuels [6, 7]; commercial terpenoids [8, 9]; polymeric compounds useful for bioplastic materials [10]; bioactive compounds and vitamins [11]; sugars [12]; and pigments with potent antioxidant activity [13]. To enable metabolic engineering in the different cyanobacterial strains, advanced genetic tools are emerging, from conventional methodologies to innovations in gene expression, genome editing and regulation systems [14–19].

CRISPR-Cas9 and -Cpf1 technologies have been successfully applied to cyanobacteria and have enabled precise genome editing, including knock-ins, knock-outs and point mutations in different genera. Briefly, Cas9 and Cpf1 proteins make a double-stranded cleavage in the genome and this break can be lethal unless it can be repaired using a suitable template [16, 20]. CRISPR technology has facilitated mutant selection and has reduced the time needed for segregation [20]. As some cyanobacteria are polyploid, it is necessary to ensure that all chromosome copies in the transformants carry identical sequences of the modified DNA [20]. Nowadays, CRISPR-based genome engineering in cyanobacteria is under active development as there are still some challenges to overcome, for instance the need to increase the repertoire of plasmids for CRISPR applications that could facilitate a suitable episomal expression for the CRISPR nuclease [19].

The first report of CRISPR-Cas9 RNA-guided genome editing system developed in cyanobacteria was carried out in *Synechococcus* 7942 in 2016 [21]. Since then, Cas9 nuclease has been used in *Synechococcus* 2973 [2, 22] and *Synechocystis* 6803 [23]. However, it has been reported that Cas9 can be highly toxic in some microorganisms and therefore other nucleases with lower toxicity have been tried to overcome this setback. An alternative to Cas9 that has been successfully developed in cyanobacteria is CRISPR-Cpf1 (Cas12a), a single RNA-guided endonuclease that, among other features, differs from Cas9 in that: i) it recognises a target with a 5′ T-rich protospacer-adjacent motif (5′-TTN-3′) instead of G-rich Cas9 PAM; ii) Cpf1-associated CRISPR arrays do not require an auxiliary trans-activating crRNA as they possess both ribonuclease activity processing the precrRNA array into mature crRNAs and nuclease activity; and iii) Cpf1 cleaves DNA via a staggered DNA double-stranded break [24, 25]. The Cpf1 derived from *Francisella novicida*, a Class II Type‐V CRISPR nuclease, has been proved to be useful for editing the genomes of *Synechocystis* 6803, *Synechococcus* 2973, *Synechococcus* 11,081 and *Anabaena* 7120 [8, 26–29]. To date, in most works, the Cpf1-CRISPR-machinery is introduced into cyanobacteria on a replicative plasmid that also includes the template for precise editing through homologous recombination [20, 30, 31].

**Fig. 1:**
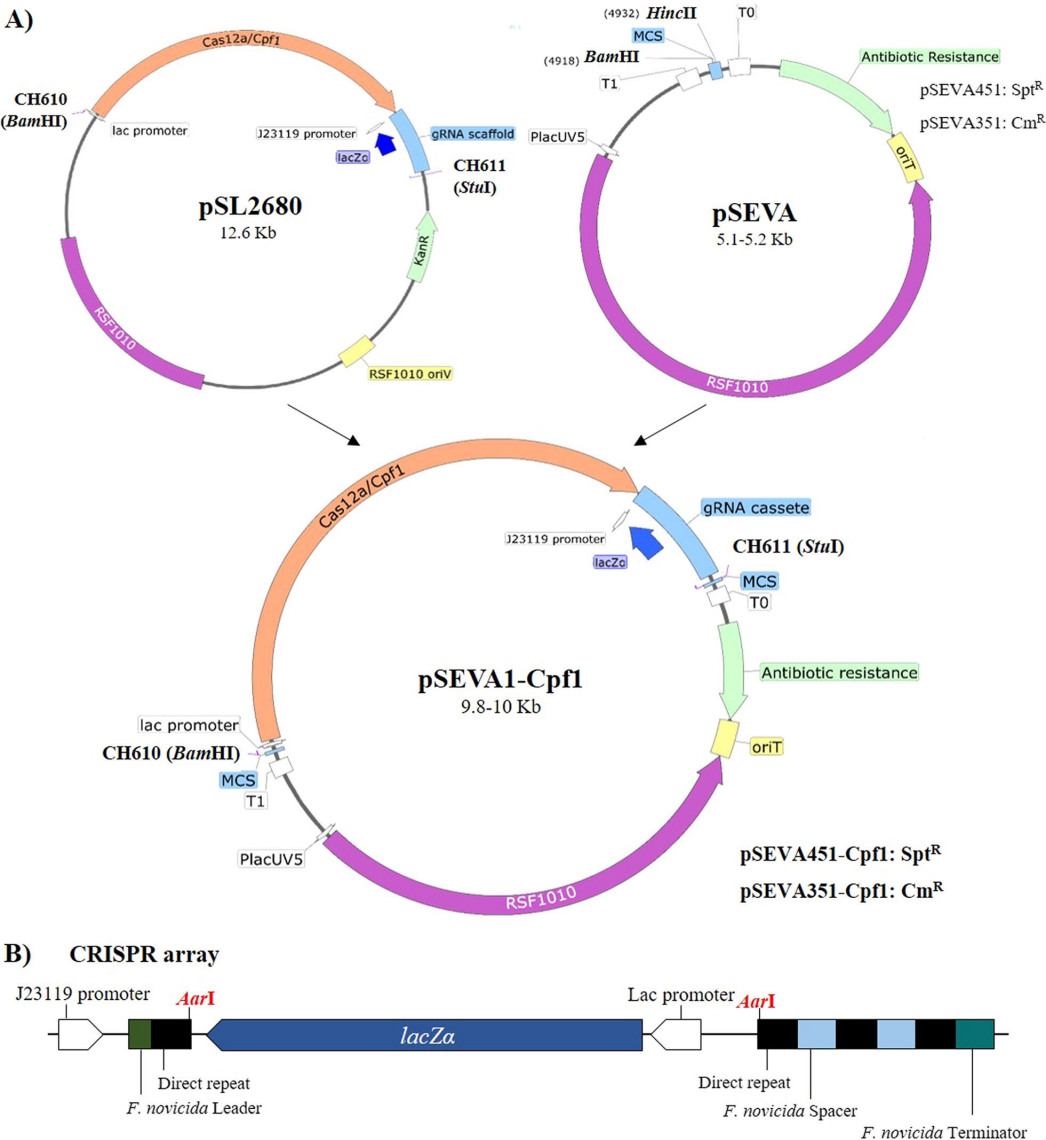
Construction and characteristics of pSEVA-Cpf1 plasmids. **A** Schematic representation of pSEVA-Cpf1 development. SEVA plasmids were modified to include the Cpf1 and CRISPR array from pSL2680. *cpf1* and CRISPR array were amplified from pSL2680 by PCR and cloned into SEVA in *Bam*HI and *Hinc*II sites. **B** Organisation of CRISPR array. *Aar*I restriction sites (red) facilitate the swapping of *lacZα* with a gRNA between two direct repeats

Ungerer and Pakrasi [28] applied the CRISPR-Cpf1 system in three different cyanobacteria: *Synechococcus*, *Synechocystis* and *Anabaena*. They used the replicative plasmid pSL2680 as a basis to construct CRISPR-Cpf1 editing plasmids (Addgene #85,581, Fig. 1A), which allows marker-less knock-ins, knock-outs and point mutations. pSL2680 contains the broad host range replicon RSF1010 that replicates in cyanobacteria and the kanamycin resistance for selection. It also contains the *Francisella novicida cpf1* nuclease gene and *Aar*I- lacZ′-*Aar*I site, flanked by CRISPR direct repeats for the cloning of the spacer sequence of crRNA. After *lacZ*, the CRISPR array keeps the two original spacers of the endogenous *Francisella novicida* CRISPR array (Fig. 1B). The expression of the CRISPR components is constitutive using a J23119 promoter (Biobrick #BBa_J23119, http://parts.igem.org/Part:BBa_J23119) for the CRISPR array and a Lac promoter for *cpf1* and *lacZα*. After the CRISPR array, a *Sal*I-*Kpn*I site could be used for the cloning of a homologous repair template [28].

Even though the authors succeeded upon using editing plasmids derived from pSL2680, they also reported that 90% of the plasmid preparation is ssDNA and therefore unclonable (Addgene #85,581, Supplementary Material). Moreover, the pSL2680 is a plasmid that makes working with it difficult since it seems to be highly unstable and have low integrity compared with other plasmids, thus one must prepare a large quantity of the plasmid for each step, which is time-consuming. Other authors have also reported problems when working with this plasmid, forcing them to introduce some modifications [26]. The CRISPR technique requires several cloning steps and it is necessary to rely on a plasmid that could yield a high quantity, with good stability and easy management that would shorten the time needed for cloning and for constructing a cyanobacterial mutant strain.

In this work, we have studied other alternatives to improve the use of CRISPR technology. pSEVA-Cpf1 plasmids have been built for the first time to have a versatile tool for genetic editing in cyanobacteria. They have been successfully tested for transformation in the common cyanobacteria *Synechocystis* 6803 and *Anabaena* 7120, and also in a non-model cyanobacterium, *Chroococcidiopsis* sp. As a proof of concept of its potential use in CRISPR technology, the deletion of the gene *nbl*A in *Synechocystis* 6803 has been obtained using the designed pSEVA-Cpf1 plasmid. The pSEVA-Cpf1 vectors developed here have a broad host range and constitute valuable tools for editing genomes, especially in cyanobacteria but also in other microorganisms.

## Results and discussion

### Assembly CRISPR-Cpf1 in a pSEVA

To improve CRISPR tools in cyanobacteria, the CRISPR-Cpf1 cassette from pSL2680 was transferred to a new and more versatile vector. For potential candidates, we chose vectors from the SEVA repository as this collection is particularly interesting owing to the modular and interchangeable structure of their plasmids [32, 33]. The Standard European Vector Architecture (SEVA, http://seva.cnb.csic.es) platform is a large repository of plasmids that are formed by three modules: an antibiotic marker, a replication origin and a cargo. These modules are separated by three permanent regions: an origin of transfer region (oriT) and two transcriptional terminators designated as T_1_ and T_0_ (Fig. 1A) [32, 34]. The SEVA platform uses a simple plasmid design that facilitates the swapping of functional modules and the extension of genome-engineering options beyond common bacterial laboratory strains [34]. Also, the SEVA vectors used here are relatively small (5100–5200 bp pSEVA351-451 respectively) compared to other vectors used in cyanobacteria, such as pPMQAK1 (8372 bp) [35], pSCB (6592 bp) [36] or pANS based vectors (7842 bp) [37], making transference easier.

SEVA vectors have been successfully used in CRISPR editing processes in gram-negative bacteria [38–40] but they have not been previously tried in gene-editing processes in cyanobacteria. So far it has been reported that in one strain, *Synechocystis* 6803, SEVA vectors containing RSF1010 or RK2 origins can be successfully transformed by natural transformation, electroporation and conjugation to express heterologous genes [41, 42]. The potential use of SEVA vectors for gene editing in cyanobacteria is therefore highly promising but it will be necessary to prove first that they can be used for transformation processes in more than one cyanobacterial strain. The SEVA vectors chosen for this study contained the RSF1010 origin that has been reported to work well on *Synechocystis* and other cyanobacteria and that have a higher copy number than those of RK2 origin (Table 1) [41, 42].

**Table 1:** Plasmids used in this study

Plasmid	Description	Reference
pSL2680	12,684 bp, RSF1010 ori, kanamycin resistance (Km.^R^), CRISPR (*cpf1* and CRISPR array)	[28]
pSEVA351	5120 bp, RSF1010 ori, chloramphenicol resistance (Cm.^R^)	[34]
pSEVA451	5334 bp, RSF1010 ori, spectinomycin resistance (Spt.^R^)	[34]
pSEVA351-Cpf1	pSEVA351 with the CRISPR machinery from pSL2680	This study
pSEVACpf1nblA	pSEVA351Cpf1 with the CRISPR array targeting *nblA* and with a homologous template for making a knock-out	This study
pSEVA451-Cpf1	pSEVA451 with the CRISPR machinery from pSL2680	This study
pRK2013	Conjugative plasmid Km.^R^, provide *tra* genes and nicking function from RK2	[43]
pRL623	Helper plasmid Cm.^R^, carries the genes for M.*Ava*I, M.*Eco*47II and M.*Eco*T22I and *mob* gene from ColK	(44)

**Fig. 2:**
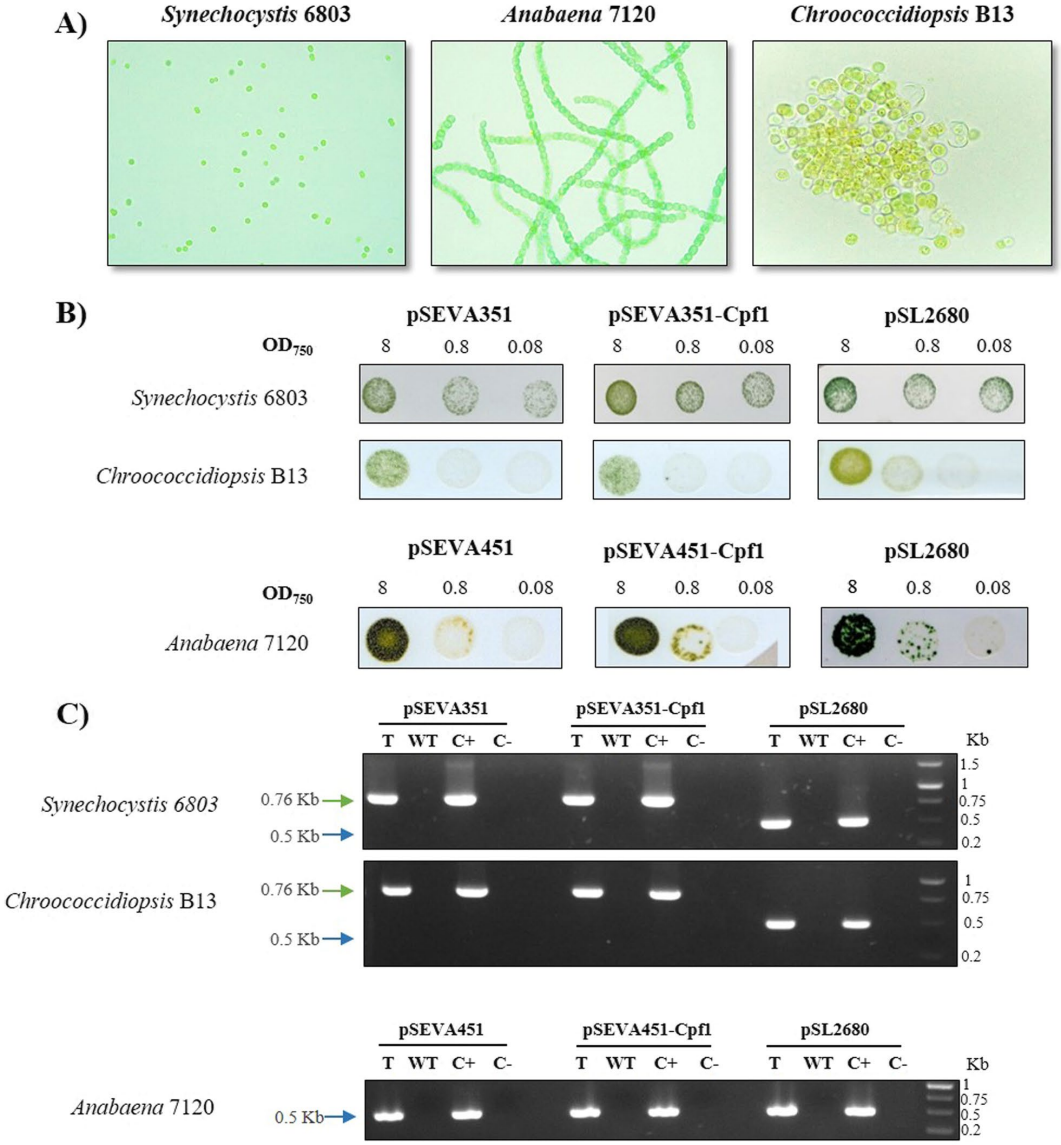
Diversity of cyanobacterial strains used in triparental mating with pSEVA, pSEVA-Cpf1 and pSL2680 plasmids.** A** Light micrographs of the cyanobacterial species used. **B** Result of the spot triparental mating for the selected cyanobacteria. Different OD_750nm_ were tested in BG11 agar with appropriate antibiotics (see Material and Methods section). **C** PCR confirmation of positive transformants. Specific primers amplifying the resistance gene in the different plasmids were used for plasmid detection (Additional file 3: Table S1). C + : PCR positive control (plasmid); T: DNA from a transformant; WT, DNA from a wild type of cyanobacteria; C-, PCR negative control (no DNA)

Then, we evaluated the use of SEVA plasmids together with pSL2680 vector in three phylogenetically diverse cyanobacterial strains with different morphologies, growth and metabolic characteristics, which are important for its potential in biotechnological applications or basic research (Fig. 2A). The unicellular *Synechocystis* 6803 was the first photosynthetic organism to have its genome sequenced. It rapidly became a model strain, being widely used in biotechnology as a photoautotrophic chassis. The second strain chosen was *Anabaena* 7120, a filamentous cyanobacterium that is interesting due to its capability to fix atmospheric nitrogen using the specialized cells called heterocyst when growing in a medium lacking combined nitrogen. Finally, we also chose a coccoidal cyanobacteria species of the genus *Chroococcidiopsis*. This genus includes extremophile strains that are a reference in the study of the resistance to desiccation, irradiation and DNA repair in cyanobacteria, among other extreme conditions [45–47]. Concretely for our study, we used *Chroococcidiopsis* sp. B13, a strain isolated from a solar panel in Spain that can resist desiccation and UV-C exposure (laboratory collection, manuscript in preparation).

Vectors were transferred into the different strains by triparental mating. *E. coli* HB101 strain bearing the conjugative plasmid pRK2013 was used as conjugative strain and HB101 harbouring the pSEVA vector was the cargo strain. pSEVA351 was used in *Synechocystis* 6803 and *Chroococcidiopsis* B13. To avoid antibiotic incompatibilities for *Anabaena* conjugation, as it needs the helper plasmid pRL623 (Cm^R^), pSEVA451 was alternatively used for this strain. As shown in Fig. 2B, all the conjugations yielded colonies after 1–2 weeks. Transformation was confirmed by the acquisition of resistance to the respective antibiotic and the presence of the plasmid was verified by PCR (Fig. 2C). pSL2680 has been used so far for genome editing in *Synechococcus* 11,801, *Synechococcus* 2973, *Synechocystis* 6803 and *Anabaena* 7120 [27–29] but this is the first successful attempt to transform this vector into a *Chroococcidiopsis* strain.

Furthermore, RSF1010 pSEVA plasmids were successfully functional in all the cyanobacteria tested (Fig. 2). Whereas most of the genetic tools for cyanobacteria reported are frequently focused on *Synechococcus* or *Synechocystis* [18], the SEVA vectors described in this work may broaden the potential applications for other strains, such as *Anabaena* and *Chroococcidiopsis* tested here and, by extension, to other cyanobacteria*.*

Next, we checked if SEVA vectors containing the CRISPR-Cpf1 endonuclease and the CRISPR array could still be transferable. To construct the pSEVA351-Cpf1 and pSEVA451-Cpf1 plasmids, the whole CRISPR system from pSL2680 was PCR-amplified and cloned into pSEVA451 or pSEVA351 (Fig. 1). The CRISPR array kept the same characteristics as the one in the original pSL2680, where the gRNA sequence targeting the gene of interest could be cloned in *Aar*I sites, replacing the *lacZα* gene. Figure 2 showed that pSEVA-Cpf1 plasmids could be transformed successfully into the three chosen cyanobacteria genera: *Synechocystis, Chroococcidiopsis* and *Anabaena*. As has been previously described for *Synechococcus* 2973 [28], no toxicity was observed in the presence of Cpf1 nuclease in all the strains tested.

Some genome editing vectors based on both Cas9 and Cpf1 have been built for *Anabaena* [26, 28, 48, 49]. On the other hand, *Chroococcidiopsis* is an extremophile strain for which just a few references relating to its genetic modification can be found but none related to CRISPR editing [50, 51]. This strain is becoming more and more relevant as it has been recently used in European Space Agency experiments due to its high resilience properties [45, 46]. Therefore, this SEVA vector opens a door to genome editing in this strain and it could be a valuable tool for researching the resistance properties to different stresses that this strain displays.

### Natural transformation of *Synechocystis* 6803 using SEVA-derived vectors

SEVA vectors containing the RSF1010 broad-host-range replicon, but harbouring different antibiotic markers (kanamycin, pSEVA251; chloramphenicol, pSEVA351; and spectinomycin/streptomycin, pSEVA451), have been successfully transformed in *Synechocystis* 6803 by natural transformation (a process in which bacteria actively take up and maintain extracellular DNA) [41]. As protocols for natural transformation are generally simple and straightforward, the use of this technique in editing processes could facilitate the rapid employment of organisms capable of natural transformation in Biotechnology [52–54]. From the three strains chosen in this work, only *Synechocystis* 6803 is able to be transformed by natural transformation, and therefore, we evaluated if the SEVA-Cpf1 vector could be also useful for natural transformation in this cyanobacterium.

**Fig. 3:**
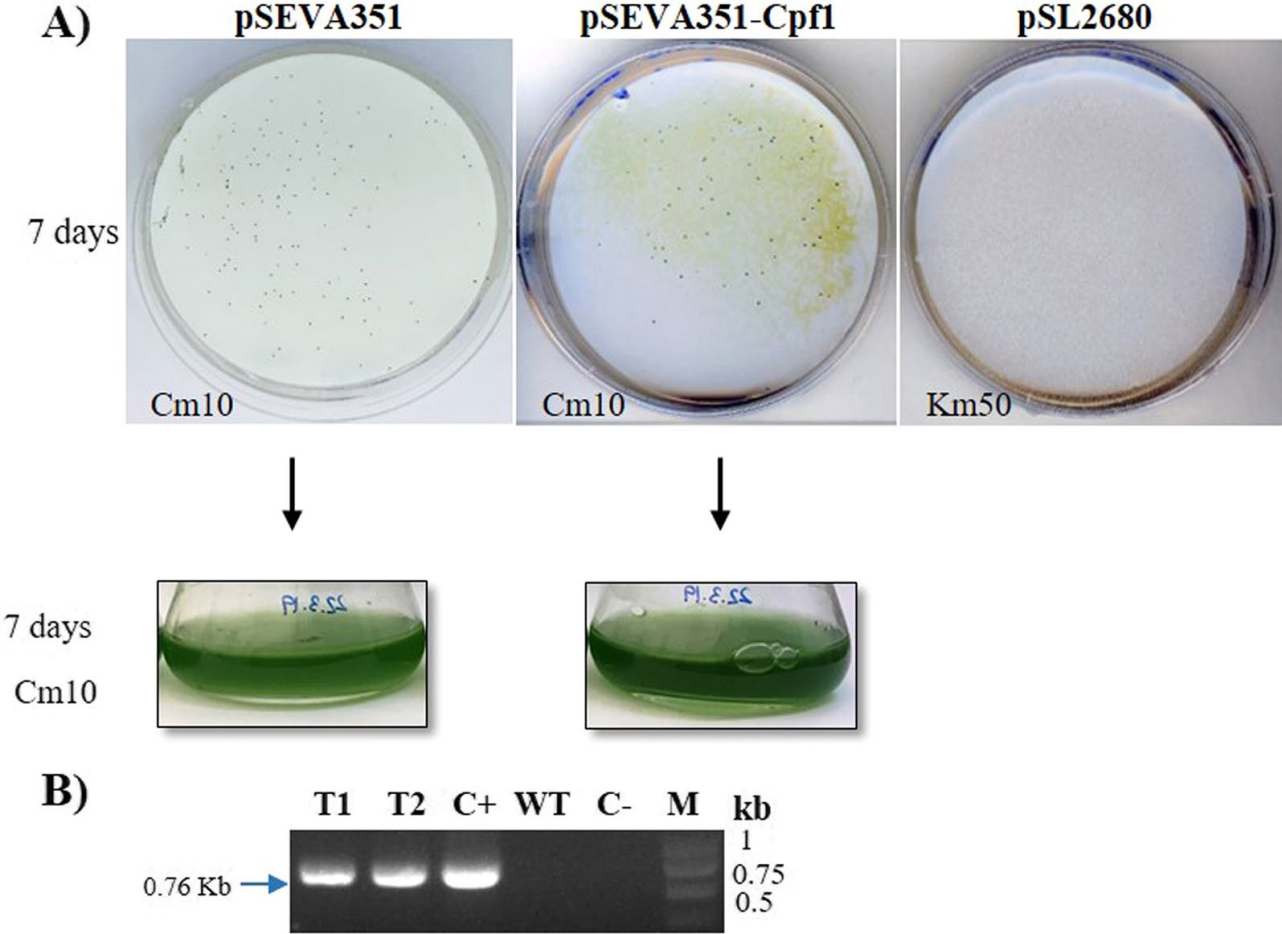
Natural transformation of pSEVA351-Cpf1 into *Synechocystis* 6803. **A** Growth of transformants after selection with appropriate antibiotics for seven days. The pSL2680 vector was not able to be transformed by natural transformation. **B** PCR confirmation of pSEVA and pSEVA351-Cpf1 presence in *Synechocystis* 6803. Specific primers amplifying the Cm resistance gene in the pSEVA and pSEVA351-Cpf1 were used for plasmid detection (Additional file 3: Table S1). The PCR results for cultures at 7 days of growth are shown. T1: DNA from *Synechocystis* 6803 with pSEVA351; T2: DNA from *Synechocystis* 6803 with pSEVA351-Cpf1; C + : plasmid pSEVA351; WT: DNA from *Synechocystis* 6803 Wild type; C-: PCR negative control (no DNA); M: molecular marker

We tested the transfer of pSEVA351-Cpf1 into *Synechocystis* 6803, using the protocol described by Sebesta et al. [55]. pSEVA351 as a positive control and pSL2680, which contains the CRISPR machinery (like pSEVA-Cpf1), were also included in the study for comparison reasons. After a week of growing on BG11 with appropriate antibiotics, transformants were obtained only when pSEVA351 and pSEVA351-Cpf1 vectors were used (Fig. 3A). Cultures of different colonies were made in BG11 with fresh antibiotics and the presence of the plasmid was verified by PCR (Fig. 3B). This experiment confirms that SEVA-Cpf1 vectors can be also used for natural transformation. On the other hand, no growth was observed in cultures transformed with pSL2680 within two weeks after transformation (data not shown). Therefore, the use of pSL2680 seems to be inconvenient for natural transformation and maybe for this reason it is advised to be used in conjugation processes instead (Addgene #85,581, supplemental material) [28].

### Markerless genomic editing of *Synechocystis* 6803 using pSEVA351-Cpf1: *nblA* gene deletion as a proof of concept

To validate the pSEVA351-Cpf1 system for genome editing processes, *Synechocystis* 6803, a cyanobacterium of industrial importance, was chosen. The genomic target selected was the deletion of the *nblA* gene coding for the non-bleaching protein A. This mutation confers an easily observable phenotype since it prevents depigmentation developed under nitrogen deprivation due to phycobilisome degradation [20]. WT phenotype bleaches under lack of nitrogen by the degradation of antenna complexes, while *nblA* mutants remain green in these conditions. For these reasons, the *nblA* gene knock-out has often been used as a reporter of editing processes in cyanobacteria [2, 20, 28].

**Fig. 4:**
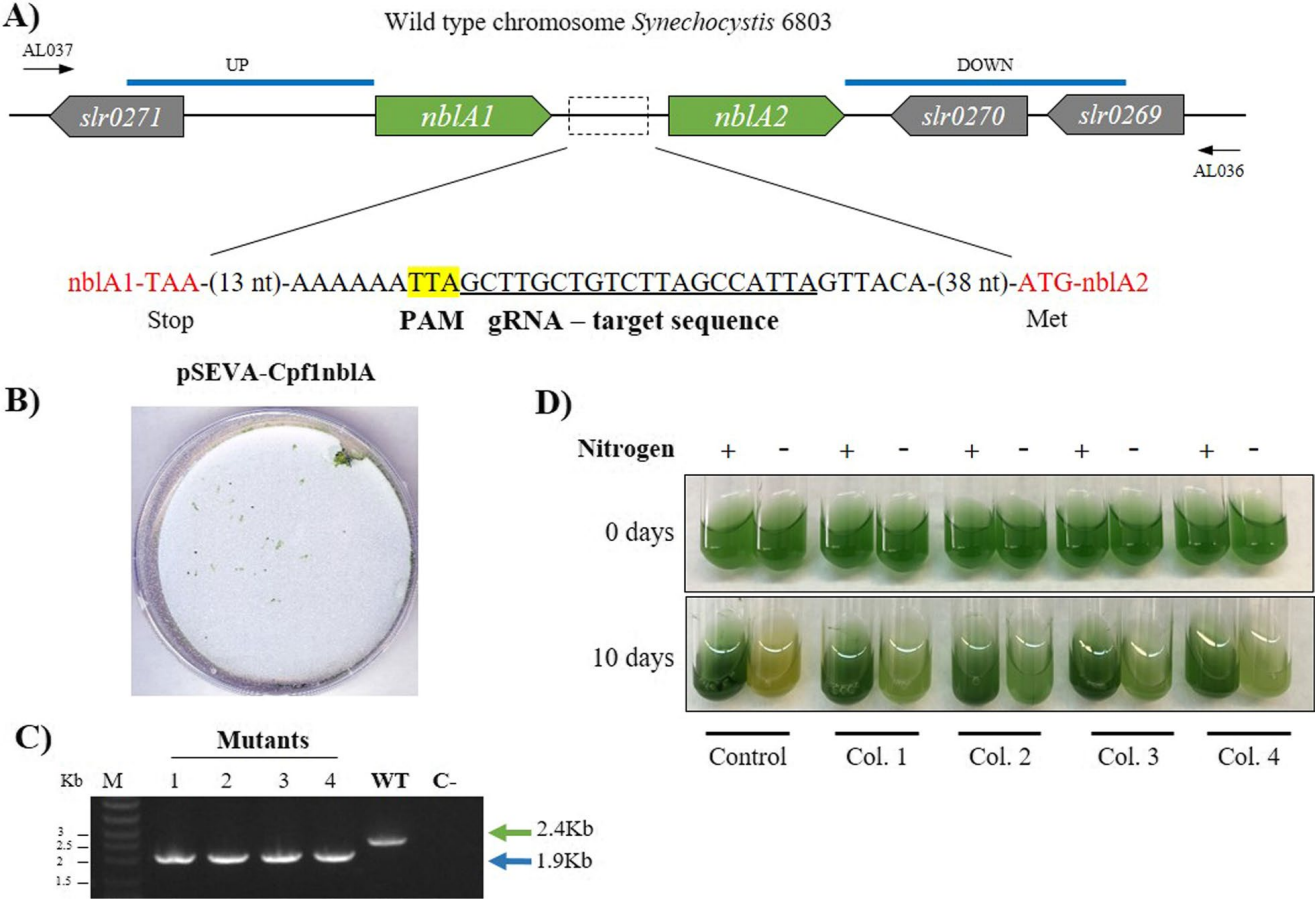
*nblA* deletion in *Synechocystis* 6803. **A** Design of CRISPR-Cpf1 genetic parts for *nblA* deletion in *Synechocystis* 6803. A scheme of the genomic context of *nblA* copies in *Synechocystis* 6803 is shown. Black arrows indicate primers used for PCR verification, blue lines indicate homology arms, green boxes indicate *nblA* genes and grey boxes the genes that surround *nblA*. The stop codon of *nblA1* and the start codon of *nblA2* are shown in red, the target sequence for the synthetic template for gRNA and the PAM sequence is also depicted. **B** Growth of colonies after natural transformation with pSEVA-Cpf1nblA plasmid. Cultures were plated in BG11 Agar Cm 10 µg/mL for pSEVA and Km 10 µg/mL for pSL2680. **C** PCR confirmation of the *nblA*1/2 deletion. The blue arrow indicates the PCR product of 4 different colonies (Lanes 1–4) when the gene has been deleted (1.9 Kb). The green arrow indicates the size of the PCR product in the wild type (WT) (2.4 Kb). C-: PCR negative control (no DNA). M: molecular marker. **D** Bleaching experiment on wild type as control and *nblA*1/2 mutant colonies (Col. 1 to 4 of the *nblA* deletion on BG11 with or without sodium nitrate)

*Synechocystis 6803* genome contains two adjacent copies of the *nblA* gene (Fig. 4A). To introduce the deletion, a synthetic template for gRNA was designed to bind between the two copies of *nblA* in a similar way as described by Ungerer and Pakrasi [28]. The directed break is subsequently repaired by double homologous recombination using a repair template cloned right after the CRISPR array (Additional file 2: Figure S2). Natural transformation was used to introduce the pSEVACpf1nblA into *Synechocystis* 6803. The colonies obtained after transformation (Fig. 4B) were submitted to three rounds of repatching onto BG11 Cm10 before analysing them by PCR (Fig. 4C). This step seems to be necessary due to the high degree of ploidy and the segregation process of *Synechocystis*. After the last patching, all the mutants selected showed a segregated selection of the *nblA*1/2 gene (Fig. 4C). In parallel, we transferred the pSEVACpf1nblA by triparental mating, following the procedure used by Ungerer and Pakrasi [28] (Additional file 2: Figure S2). Colonies appeared after 7 days and they were also patched three times onto BG11 with Cm. PCR confirmed the deletion segregation in these mutants (Additional file 2: Figure S2B). The sequencing of these PCR amplicons (from edited colonies transformed by natural transformation and conjugation) confirmed the expected deletion.

We proceeded to test the non-bleaching phenotype of the *nblA* mutant’s deletion obtained after 3 patches on selective media. To further verify segregation, we performed bleaching tests using wild-type and the selected Δ*nblA Synechocystis* 6803. After 10 days of additional growth, qualitative differences in colour were registered as shown in Fig. 4D (natural transformation) and Additional file 2: Figure S2C (triparental mating). The mutant colonies of *Synechocystis* 6803 did not display the colour of the WT that was bleached upon removal of nitrate, indicating that they were fully segregated for the *nblA* deletion. Therefore, pSEVA-Cpf1 is useful for editing processes on *Synechocystis* and it could also be useful for editing processes on other cyanobacteria.

We have proved that pSEVA-Cpf1 vectors can be used in the transformation of several genera of cyanobacteria. Using pSEVA-Cpf1, we successfully edited the *Synechocystis* 6803 genome by deleting the two copies of *nblA*. Moreover, the pSL2680 failed when using natural transformation but not the pSEVA vectors, which could be assimilated by this procedure even when the genetic editing of *Synechocystis* 6803 was carried out. Apart from *Synechocystis* 6803, in this work we have shown for the first time the replication of pSEVA-Cpf1 vectors in two cyanobacterial species different from those previously reported: *Anabaena* 7120 and *Chroococcidiopsis* sp. These results demonstrate that pSEVA and derived vectors are a promising tool for the transformation of other biotechnological relevant cyanobacteria, for instance extremophile bacteria. Considering the SEVA design, our plasmids would broaden the scope of cyanobacteria genera able to be CRISPR edited. Additionally, SEVA vectors could be also the basis for further development of CRISPR technology, e.g. they could be modified to include inducible CRISPRi gene repression elements.

What is more, if no CRISPR-mediated gene editing were pursued, considering the modular architecture of SEVA vectors, different combinations of promoters, gene reporters or the chance to clone any desired gene into the SEVA backbone would provide a source of multiple possibilities for obtaining genetically modified cyanobacteria. Additionally, as pSEVA has shown to be functional in other bacteria, these plasmids could be transferred to different microorganisms, increasing the synthetic biology tools available.

**Fig. 5:**
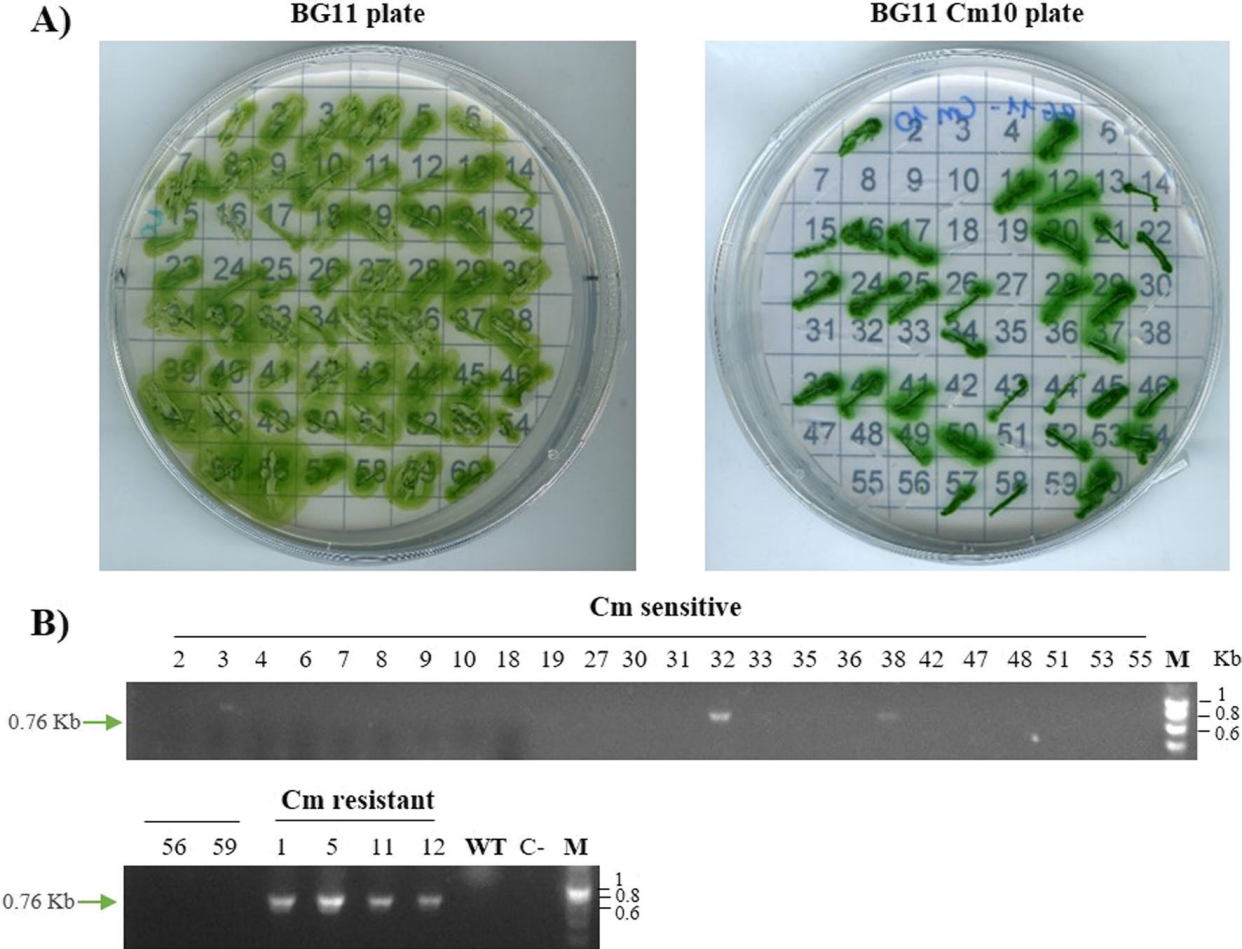
pSEVA351-Cpf1nblA curing in *Synechocystis* 6803.** A** Streak of edited colonies on BG11 antibiotic-free medium and BG11 Cm10 plates 12 days growth. **B** PCR confirmation of pSEVA351-Cpf1nblA presence in *Synechocystis* 6803. Specific primers amplifying the Cm resistance gene were used (Additional file 3: Table S1). All the Cm sensitive colonies were analyzed by PCR (Cm sensitive). As controls, four Cm resistant colonies (Cm resistant) were included. WT: DNA from *Synechocystis* 6803 Wild type; C-: PCR negative control (no DNA); M: molecular marker

In order to allow more than one round of genome editing, the plasmid must be cured unless the next editing vector contains a different selection marker. Therefore, we cured the pSEVA-Cpf1nblA plasmid from an *nblA Synechocystis* 6803 colony. A patch of an edited strain was grown on BG11 plate without antibiotics for one week, then a sterile pipette tip was deeped on the patch and cells were resuspended in 4 mL BG11 and cultured to an OD_750_ of 1 to allow spontaneous plasmid loss. In order to obtain single cells, the culture was serially diluted and plated on BG11. Sixty single colonies were picked and streaked on BG11Cm10 and BG11 plates to identify colonies that had become sensitive to chloramphenicol, therefore, they have lost the editing plasmid (Fig. 5A). Finally, the absence of plasmid was checked by PCR (primers oligo 7/8,Additional file 3: Table S1) in all chloramphenicol sensitive colonies (Fig. 5B). Of the 60 clones tested, 26 were sensitive to chloramphenicol (Figura 5A), but only 2 of the colonies that had lost resistance to the antibiotic still showed the presence of the plasmid when analyzed by PCR. Since a colony includes several cells generations, plasmid loss could still be an undergoing process in those two mutants. These results demostrated that editing plasmid was lost in 40% of the colonies for the *nblA* deletion, 5 times more than the 8% cured colonies for the *nblA* deletion reported in *Synechococcus* 2973 using a pSL2680 derived plasmid [28]. Up to date, to remove plasmids, cells are grown in an antibiotic-free medium for many generations which is time consuming. Recently Niu et al. [26] described the use of the counter selection marker *sacB,* which encodes the *Bacillus subtilis* levansucrase [56] for curing the pCpf1 editing vector (a pSL2680-derived plasmid) in *Anabaena*. In the presence of 5% sucrose, *sacB* expression is lethal and therefore, survival depends on recombination events or alternatively on the spontaneous loss of the *sacB*-containing vector, allowing the curation of the editing plasmids from cyanobacteria cells [26, 57]. Our results indicate that there is not need of an additional selective marker to obtain a hight percentage of cured colonies, making easier a further edition step.

## Conclusions

In this work, we have proved that RSF1010 SEVA vectors harbouring the CRISPR-Cpf1 system are suitable for transforming different genera of cyanobacteria: from industrial and research strain models, e.g. *Synechocystis* or *Anabaena,* to non-model extremophiles such as *Chroococcidiopsis*. SEVA-based vectors can be also transferred by natural transformation into the model *Synechocystis* 6803. As a proof of concept of the potential of pSEVA-Cpf1 vectors in CRISPR-editing processes, we have successfully applied this vector for a markerless genomic editing in *Synechocystis* 6803, deleting the *nblA* reporter gene. Thus, this system could overcome the hurdle of using other Cpf1-editing vectors such as pSL2680, whose disadvantages include needing higher quantities for cloning, a less versatility and a low rate of cured colonies.

To summarise, our study proves that SEVA-based plasmids can be efficient for editing processes in cyanobacteria. Their simplicity, their modular structure that allows an easy interchange of different cargos, their small size and their free cost make these vectors an intelligent and robust alternative for use in cyanobacteria. We anticipate that the use of the optimised SEVA editing systems will not only contribute towards expanding the molecular toolbox of cyanobacteria but may also facilitate the development of other biotechnologically relevant microorganisms, such as extremophiles.

## Materials and methods

### Bacterial strains, media and culture conditions

Cloning steps were performed in *Escherichia coli* strain DH5α while *Escherichia coli* strain HB101 was used for conjugation. *E. coli* cells were grown at 37 °C in liquid LB or on LB agar plates supplemented with 50 μg/mL kanamycin, 10 μg/mL chloramphenicol, or 50 μg/mL spectinomycin as required. *Synechocystis* 6803 and *Anabaena* 7120 were a kind donation from Dr Luis López-Maury (IBVF-CSIC, Seville, Spain). *Chroococcidiopsis* was isolated from a solar panel and characterised in our laboratory (laboratory collection, manuscript in preparation). *Synechocystis* 6803, *Anabaena* 7120 and *Chroococcidiopsis* B13 cells were cultivated in BG11 (blue-green 11) medium, a freshwater standard growth medium for cyanobacteria with the following composition: 1.5 g/L NaNO_3_; 0.02 g/L Na_2_CO_3;_ 0.03 g/L K_2_HPO_4_; 0.075 MgSO_4_ * 7 H_2_O; 0.036 g/L CaCl_2_ * 2 H_2_O; 1 mg/L Na_2_EDTA * 2 H_2_O; 1.81 mg/L MnCl_2_* 4 H_2_O; 0.05 mg/L CoCl_2_* 6 H_2_O; 0.039 mg/L Na_2_MoO_4_*H_2_O; 0.08 mg/L CuSO_4_* 5 H_2_O; 0.22 mg/L ZnSO_4_ * 7 H_2_O; 2.86 mg/L H_3_BO_3_; 6 mg/L citric acid and 6 mg/L ferric ammonium citrate (PhytotechLabs). BG11 medium was buffered to pH 7.5 with 10 mM HEPES. Cyanobacteria were grown on 1.5% agar plates or liquid medium at 30 °C under 100 μmol photon • m^−2^ • s^−1^ of continuous white light. For mutant strains growth, appropriate antibiotics were added. Chloramphenicol 10 μg/mL was used for pSEVA351 and pSEVA351-Cpf1 selection in both *Synechocystis* and *Chroococcidiopsis*; kanamycin 50 μg/mL and 150 μg/mL was used for *Synechocystis* and *Chroococcidiopsis* selection after pSL2680 conjugation. Spectinomycin 10 μg/mL was used for *Anabaena* in pSEVA451 and pSEVA451-Cpf1 conjugations; and for pSL2680 *Anabaena* conjugation neomycin 50 μg/mL was used.

The primers used in this study are listed in Additional file 3: Table S1. T4 DNA ligase, In-fusion HD cloning kit and all restriction enzymes were from Takara, except *Aar*I, which was purchased from Thermo Fisher Scientific. KOD-Hot start polymerase (Novagen) was used for high-fidelity polymerase chain reaction (PCR). The plasmid miniprep and DNA purification kits were obtained from Nzytech. Oligonucleotides were synthesized by Condalab. All protocols were conducted according to the manufacturer’s instructions. Plasmids maps were created with SnapGene 3.1 (GSL Biotech LLC).

### Conjugation of plasmids into *Synechocystis* 6803, *Chroococcidiopsis* sp. B13 and *Anabaena* 7120

The conjugation of pSEVA and CRISPR-Cpf1 plasmids into all strains was performed following the spot mating protocol of Elhai and Wolk [43]. *E. coli* HB101 strain bearing the conjugative plasmid pRK2013 was used as a conjugative strain; on the other hand, HB101 strain bearing different plasmids (cargo strain) pSEVA351/451, pSEVA-Cpf1 or pSEVACpf1nblA was used, depending on the experiment. In *Synechocystis* 6803 and *Chroococcidiopis* B13 conjugations cultures of OD_750_ 2–3 were used. For conjugation into *Anabaena* 7120, the cargo strain also contained the helper plasmid pRL623. Prior to mating, *Anabaena* cultures were prepared as previously described [35] with a few modifications. *Anabaena* 7120 cultures of OD_750_ 1–2 were disrupted by sonication with a P-Selecta Ultrasens bath until the average filament length was 6–8 cells long, determined by visual assessment with a microscope. For recovery, the sonicated cells were incubated at 30 ºC without shaking under low-light conditions for 6 h, then the culture was collected by centrifugation at 4000 rpm for 10 min at 15 ºC. For the three cyanobacteria strains, OD_750_ was adjusted from 14 to 0.008 in order to test different proportions of cyanobacteria:*E. coli* for mating. Then the cyanobacteria were mixed with *E. coli* and plated onto BG11 5% LB (vol/vol) agar Plates were incubated for 48 h at growth conditions. After that time, *Synechocystis* 6803 and *Chroococcidiopsis* sp. B13 conjugation filters were transferred onto BG11 agar supplemented 10 μg/mL Chloramphenicol (pSEVA plasmids) and with 50 μg/mL or 150 μg/mL kanamycin (pSL2680), respectively. Colonies appeared after 10 days for *Chroococcidiopsis* sp. B13 and 7 days for *Synechocystis* 6803. After 48 h, *Anabaena* 7120 conjugation filters were transferred onto BG11 agar supplemented with 10 μg/mL spectinomycin (pSEVA vectors) or 50 μg/mL neomycin (pSL2680) and colonies appeared within 7 days.

### Natural transformation of *Synechocystis* 6803

*Synechocystis* natural transformation was carried out as previously published [55] with a few modifications. Briefly, *Synechocystis* 6803 was grown to mid-log phase (OD_750_≈0.7) and, for each transformation, 10 mL of cell culture was centrifuged at 4000 rpm 20ºC for 5 min. The supernatant was discarded, and the cell pellet was suspended in 100μL of BG11. For each transformation, 1 μg of the plasmid was used. In addition to the tubes destined for transformation, a no-DNA negative control was also prepared. After 5 h of incubation at 30ºC, cells were plated onto BG11 without antibiotics and incubated at 30ºC for 2 days. Then, filters were transferred onto BG11 with appropriate antibiotics. After approximately 7 days, the transformants were inoculated in a flask with fresh antibiotics, left to grow for a week and then screened for plasmid presence using PCR.

### Construction of pSEVA-Cpf1 and pSEVA-Cpf1RNA plasmids

To construct the pSEVA351-Cpf1 and pSEVA451-Cpf1 plasmids, the whole CRISPR system from pSL2680 was PCR-amplified using CH610 and CH611 primers. The amplicon was digested with *Bam*HI and *Stu*I and cloned into pSEVA451 or pSEVA351 digested with *Bam*HI and *Hin*cII.

pSL2680 and therefore pSEVA-Cpf1 plasmids include a modification of the native CRISPR array of *Francisella novicida*, which has three spacers. The first spacer has been replaced by *LacZ* flanked by *Aar*I recognition sites to allow gRNA cloning, but the second and third are endogenous native of *Francisella novicida* to keep its natural structure to allow a correct gRNA processed fragment (Ungerer and Pakrasi, 2016). The assembly of the synthetic template for gRNA-*nblA* in the pSEVA-Cpf1 vector was carried out as follows. pSEVA351-Cpf1 was digested for 4 h with *Aar*I at 4 U/µg and 1 μM of the provided oligonucleotide (Thermo Fisher). The template for gRNA was constructed by annealing the 5’-phosphorylated primers AL001 and AL002 in ligation buffer (Takara). Primers used had 4 nucleotide overhangs compatible with *Aar*I digestion in the plasmid. The reaction was cooled slowly using the following program: 95 °C 3 min, 95 °C 2 min, cool to 55ºC at 0.1ºC/sec, 50ºC 5 min, cool to 22 ºC at 0.1 ºC/sec, 22 ºC 2 min. The gRNA template was then diluted 1/20 and ligated to the digested plasmid to yield pSEVACpf1RNA. In that plasmid, the gRNA targeting *nblA* replaces the *lacZ* in the CRISPR array, but keeps the two original spacers of the natural *Francisella novicida* CRISPR array (Fig. 1B). The processing of the pre-crRNA from CRISPR array transcription results in the gRNA of interest (from the first spacer) and other two, with no targets in the cyanobacteria.

After cloning the gRNA, a PCR with the pair of primers AL004/AL016 and AL005/AL015, followed by an overlap PCR with AL015/AL016, was used to obtain the up and bottom homology arms (~ 1000 bp each one) of the editing template. These arms correspond to the flanking DNA surroundings of the *nblA* genes and they both combined will be used as the repair template after the double-strand break. The resulting PCR fragments were cloned using an In-fusion HD cloning kit into pSEVACpf1RNA digested with *Pst*I to yield the plasmid pSEVACpf1nblA (Additional file 1: Figure S1).

### PCR to confirm plasmid transformation and accurate editing

Rapid DNA extraction with chloroform was realised for each transformant and PCR was performed to verify the plasmid transformations and *nblA* deletion. NZYTaq II 2 × Green Master Mix (Nzytech) was used for all PCR confirmation reactions. The set composed of Oligo 7/Oligo 8 was used to check for Cm^R^ gene in pSEVA351 and derived plasmids, the AL040/AL041 set was used to check for the Spt^R^ gene in pSEVA451 and derived plasmids, the AL034/AL035 set was used to check for the Km^R^ gene in pSL2680 and derived plasmids, and the AL036/AL037 set was used to check for the deletion of *nblA* on *Synechocystis* 6803 chromosome. In *nblA* edited strains, PCR fragments were purified using NZYGelpure kit (Nzytech) and sequenced to confirm the accurate edition (Eurofins Genomics).

### Bleaching test under nitrogen deprivation conditions

The WT and mutants were inoculated into 4 mL of BG11 at 30 °C under 100 μmol photon • m^−2^ • s^−1^ of continuous white light and 150 rpm until late linear growth. Then cultures were washed 3 times with 30 mL BG11_0_ (BG11 without sodium nitrate) and used to start fresh cultures in BG11_0_ at an OD_750_ of 2. They were grown for 10 days before registering qualitative differences in colour.

## Supplementary Information

**Additional file 1:** **Figure S1.** pSEVACpf1nblA plasmid used for nblA deletion in Synechocystis 6803. It contains the cpf1, a synthetic template for a gRNA targeting nblA and a homologous repair template for deletion

**Additional file 2:** **Figure S2. **Conjugation of pSEVACpf1nblA into *Synechocystis* 6803 for *nblA* deletion. A) Growth of colonies after conjugation with pSEVA-Cpf1nblA plasmid. Cultures were plated onto BG11 agar supplemented with Cm 10µg/mL (see Material and Methods section) **C)** PCR confirmation of the *nblA*1/2 deletion. The blue arrow indicates the PCR product of 4 different colonies (Lanes 1-3) when the gene has been deleted (1.9Kb). The green arrow indicates the size of the PCR product in the wild type (WT) (2.4Kb). C-: PCR negative control (no DNA). M: molecular marker. **C)** Bleaching experiment on wild type as control and *nblA*1/2 mutant colonies (Col. 1 to 4 of the *nblA* deletion on BG11 with or without sodium nitrate).

**Additional file 3**: **Table S1.** Primers used in this study

## Data Availability

All data generated or analysed during this study are included in this published article and its supplementary information files.

## Abbreviations

Cas: CRISPR associated protein
CRISPR: Clustered regularly interspaced short palindromic repeats
PCR: Polymerase chain reaction
gRNA: Synthetic guide RNA
