# Supplementary figures and images for: SEVA-Cpf1, a CRISPR-Cas12a vector for genome editing in cyanobacteria
Source: Microb Cell Fact. 2022 May 28;21:103. doi: 10.1186/s12934-022-01830-4 (PMC9148489; doi:10.1186/s12934-022-01830-4)

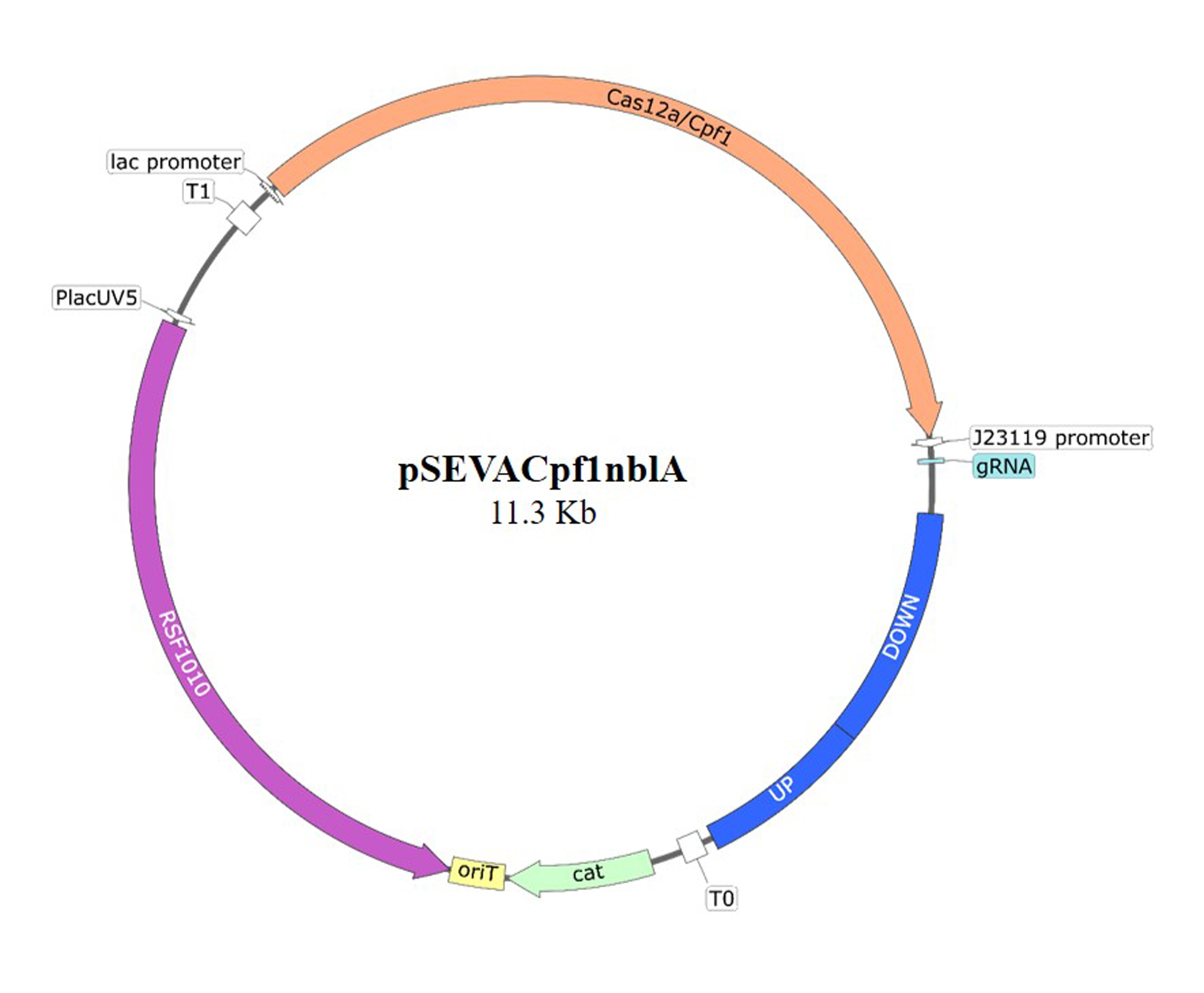

Supplement: Supplementary file 1 — Additional file 1: Figure S1. pSEVACpf1nblA plasmid used for nblA deletion in Synechocystis 6803. It contains the cpf1, a synthetic template for a gRNA targeting nblA and a homologous repair template for deletion [file 12934_2022_1830_MOESM1_ESM.jpg]

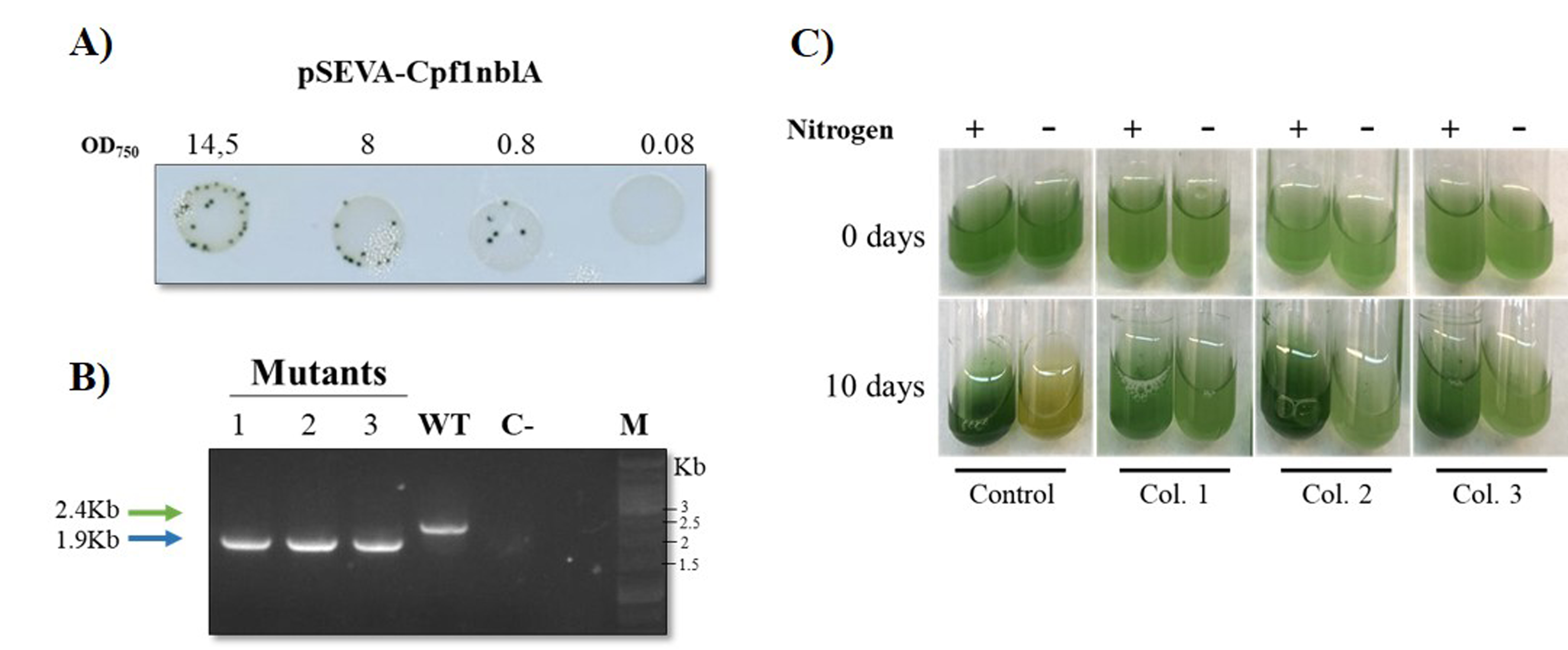

Supplement: Supplementary file 2 — Additional file 2: Figure S2. Conjugation of pSEVACpf1nblA into Synechocystis 6803 for nblA deletion. A) Growth of colonies after conjugation with pSEVA-Cpf1nblA plasmid. Cultures were plated onto BG11 agar supplemented with Cm 10µg/mL (see Material and Methods section) C) PCR confirmation of the nblA1/2 deletion. The blue arrow indicates the PCR product of 4 different colonies (Lanes 1-3) when the gene has been deleted (1.9Kb). The green arrow indicates the size of the PCR product in the wild type (WT) (2.4Kb). C-: PCR negative control (no DNA). M: molecular marker. C) Bleaching experiment on wild type as control and nblA1/2 mutant colonies (Col. 1 to 4 of the nblA deletion on BG11 with or without sodium nitrate). [file 12934_2022_1830_MOESM2_ESM.jpg]
