# Supplementary material for: SEVA-Cpf1, a CRISPR-Cas12a vector for genome editing in cyanobacteria
Source: Microb Cell Fact. 2022 May 28;21:103. doi: 10.1186/s12934-022-01830-4 (PMC9148489; doi:10.1186/s12934-022-01830-4)
Supplement: Supplementary file 3 — Additional file 3: Table S1. Primers used in this study [file 12934_2022_1830_MOESM3_ESM.docx]

Table S1. Primers used in this study

| **Name** | **Sequence** | **Use** |
| --- | --- | --- |
| CH610 | **CTCGAGACTAGTGGATCC**TCGATGTAACCCACTCGTGC | PCR cassette Cpf1 CRISPR array of pSL2680. Restriction sites are marked in bold. |
| CH611 | **TACGTAAGTACTAGGCCT**TCTAGAGTCGACGGTACCAA |  |
| AL001 | 5P-AGATGCTTGCTGTCTTAGCCATTA | Guide RNA targeting *nblA* |
| AL002 | 5P-AGACTAATGGCTAAGACAGCAAGC |  |
| AL004 | GATGCCTAAACCTAGAGTTGAGCTGTTGCCCTCCAAGG | Overlapping primers for *nblA* deletion |
| AL005 | CCTTGGAGGGCAACAGCTCAACTCTAGGTTTAGGCATC |  |
| AL015 | **ACTCTAGAAGGGACC**CACCCAAGGAAATGCATTACTTAC | Infusion cloning in pSEVA. Homologous region is shown in bold. |
| AL016 | **GCAAGCTTGCATGCC**GATAGCCCTGAGCACCAGAAG |  |
| AL034 | GGTAGCGTTGCCAATGATGT | Plasmid pSL2680 verification (Km^R^ gene) |
| AL035 | GGCAAGATCCTGGTATCGGT |  |
| AL036 | CAATCTCTACCTAGCTCTGACA | *nblA* deletion verification |
| AL037 | TTGTTGGGATGTATAACCCTGG |  |
| Oligo 7 | TGATCGGCACGTAAGAGGTTCC | Plasmid pSEVA351 verification (Cm^R^ gene) |
| Oligo 8 | TTACGCCCCGCCCTGCCACT |  |
| AL040 | ATGCCTCGGGCATCCAAGCA | Plasmid pSEVA451 verification (Spt^R^ gene) |
| AL041 | TCAGCAAGATAGCCAGATCA |  |
